# Supplementary material for: Fast automatic multiscale electron tomography for sensitive materials under environmental conditions
Source: Commun Eng. 2025 Aug 11;4:149. doi: 10.1038/s44172-025-00482-7 (PMC12340152; doi:10.1038/s44172-025-00482-7)
Supplement: Supplementary file 2 — Supplementary Information [file 44172_2025_482_MOESM2_ESM.pdf]

Fast automatic multiscale electron tomography for sensitive materials under environmental

Supplementary information

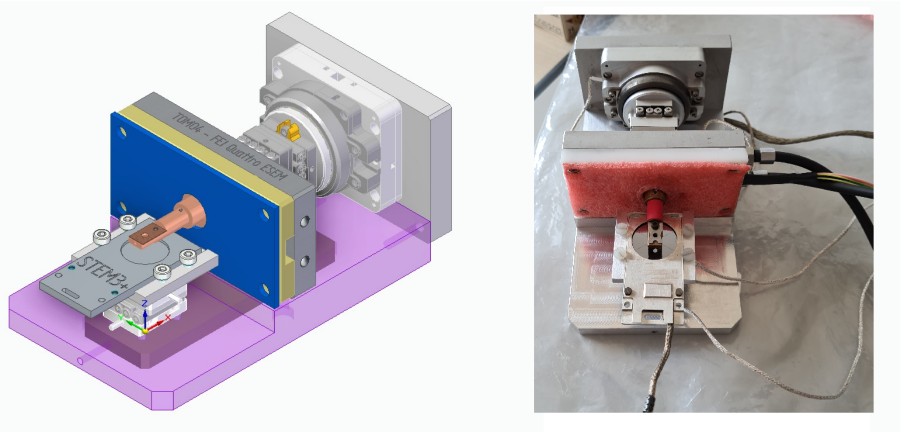

Supplementary Figure 1 Figure showing the 3D CAD model and the photo of the home-made "Tomo 4" tomographic ESEM stage. The stage consists of a base, a Peltier module to control the temperature at the tip of the sample holder and piezo-inertial elements to move the tip. The STEM3+ detector is placed on other piezo-inertial elements to control the position of the detector under the sample.

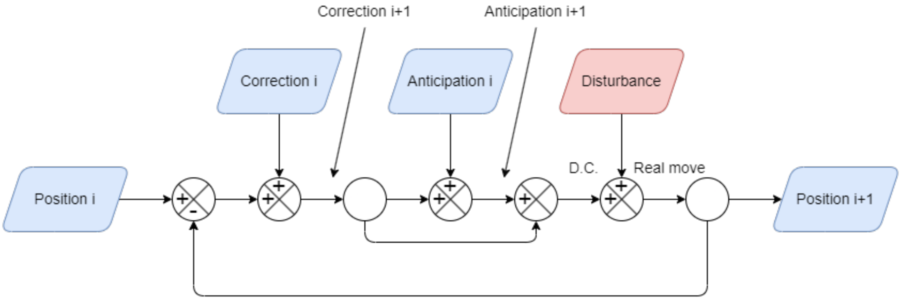

Supplementary Figure 2 Block diagram of the predictive *in situ* drift correction algorithm between step n and $n+1$. D.C. stands for Drift Correction.

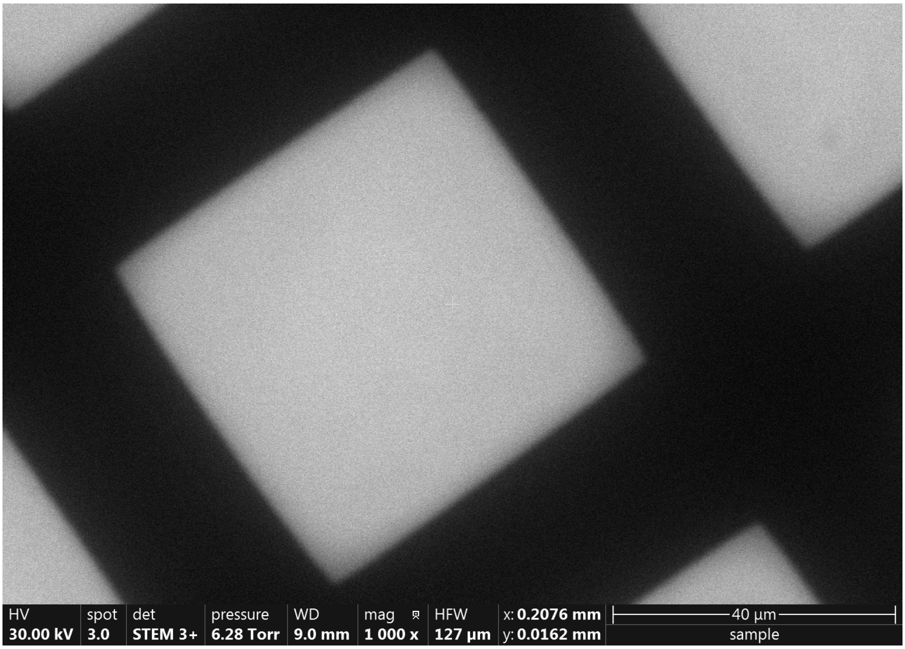

Supplementary Figure 3 Projection in ADF mode of one of the TEM grid squares just after the beam is switched on. The blurred white zone is characteristic of a thick layer of water. By reducing the pressure and keeping the temperature stable, it is possible to make the sample transparent to electrons.

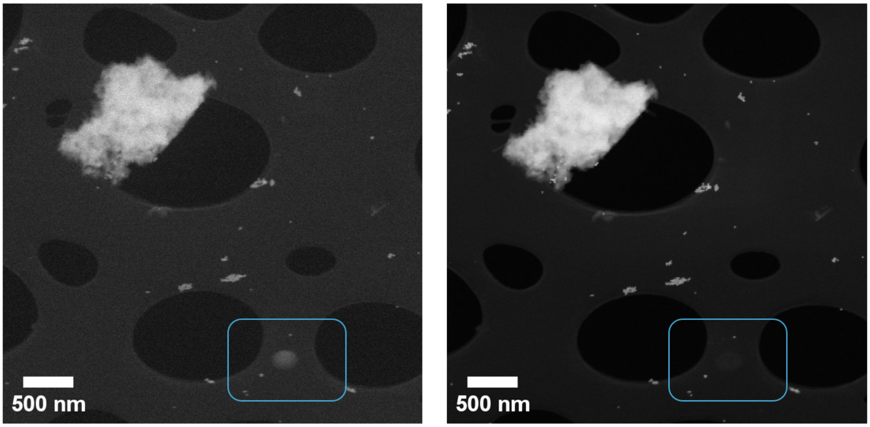

Supplementary Figure 4 Figure showing the environment close to the sample during the first and second series of tilts. The projection on the left corresponds to the first tilt series and shows a nanodroplet of water with a partial hemispherical shape. In the projection on the right, corresponding to the second tilt series, the droplet has evaporated and only dry residue remains, leaving a stain.

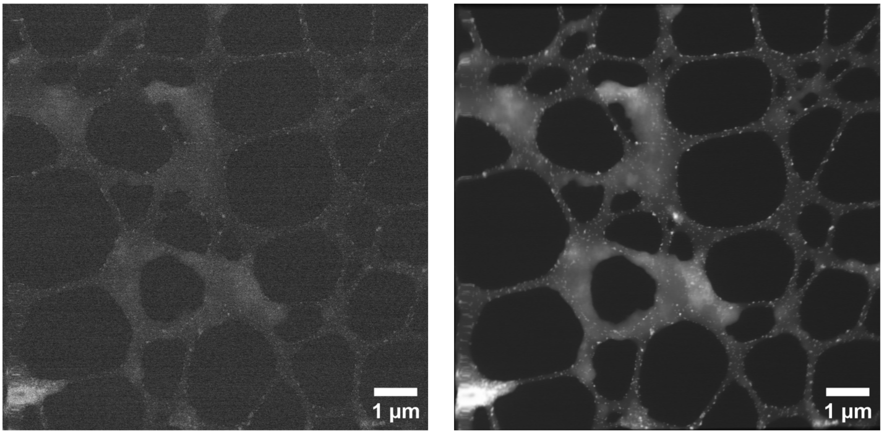

Supplementary Figure 5 Figure of the environment close to the sample after temperature and pressure stabilisation, before tilt series acquisition. The projection on the left corresponds to the raw projection. The projection on the right shows the denoising of the same (N2V algorithm), with training including other projections from the same experiment. In both projections, the large uniform grey areas indicate the presence of liquid water.

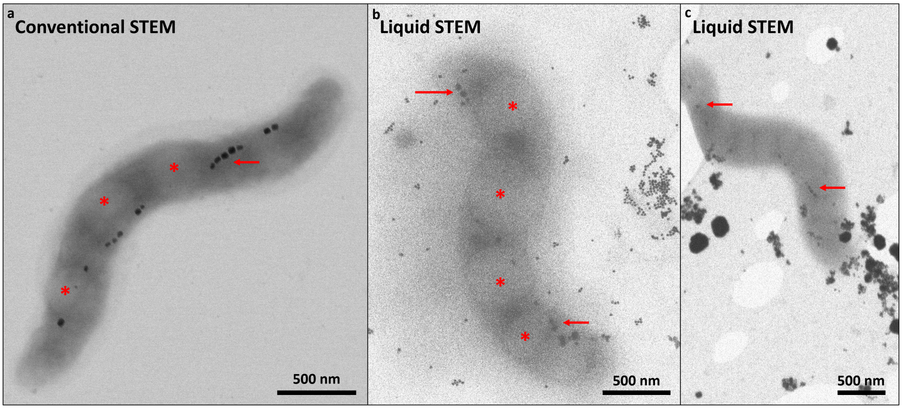

Supplementary Figure 6 Electron micrographs of AMB-1 bacteria obtained from conventional (dry sample) and liquid STEM. Red arrows and stars point to the magnetite chains and PHA granules in the bacteria, respectively. These intracellular structures are preserved in hydrated cells (liquid STEM).
